# Microbiome‐mediated chemical communication in insects: Implications for pest management

**DOI:** 10.1002/ps.71015

**Published:** 2026-06-12

**Authors:** Ioannis Eleftherianos, Wei Zhang, Amr Mohamed, Rasha K. Al‐Akeel, Abeer M. Alkhaibari, Nemat Keyhani, Guy Smagghe

**Affiliations:** ^1^ School of Biological Sciences, Institute for Global Food Security Queen's University Belfast Belfast UK; ^2^ State Key Laboratory of Green Pesticide Guizhou University Guiyang China; ^3^ Department of Entomology, Faculty of Science Cairo University Giza Egypt; ^4^ Department of Zoology, Faculty of Science King Saud University Riyadh Saudi Arabia; ^5^ Department of Biology, Faculty of Science University of Tabuk Tabuk Saudi Arabia; ^6^ Department of Biological Sciences University of Illinois Chicago IL USA; ^7^ Institute of Entomology Guizhou University Guiyang China; ^8^ Department of Biology Vrije Universiteit Brussel (VUB) Brussels Belgium

**Keywords:** insect microbiome, semiochemicals, chemical communication, volatile organic compounds, pest management

## Abstract

Insects rely on semiochemicals to regulate aggregation, mating, foraging, and host selection. This review synthesizes evidence that insect‐associated microbiota shape these chemical signals and evaluates their potential for pest management. The literature supports four principal routes by which microbes influence insect chemical communication: (i) direct production of volatile organic compounds; (ii) microbial provision or modification of pheromone precursors; (iii) microbiome‐mediated effects on sensory and neural function; and (iv) context dependence driven by diet, development, quorum sensing, and environmental microbiomes. The strongest evidence comes from loss–gain–rescue studies, including German cockroach fecal volatiles and *Wolbachia*‐linked effects on *Drosophila paulistorum* mating chemistry. Translationally, fermentation‐based lures for fruit flies and microbial deterrents such as *Xenorhabdus*‐derived metabolites show clear promise, whereas most other systems remain correlative because gene‐to‐metabolite‐to‐behavior chains are incomplete and field validation is limited. Microbiome–semiochemical interactions offer a credible platform for next‐generation attractants, repellents, and symbiont‐based pest control. Progress will depend on rigorous mechanistic validation, standardized behavioral assays, and biosafety‐aware field testing to convert promising discoveries into scalable integrated pest management tools. © 2026 The Author(s). *Pest Management Science* published by John Wiley & Sons Ltd on behalf of Society of Chemical Industry.

## Graphical abstract

Microbiome–semiochemical interactions involve the following processes: direct microbial synthesis, host gene regulation, precursor biotransformation, microbiome modulation and indirect ecological signaling.

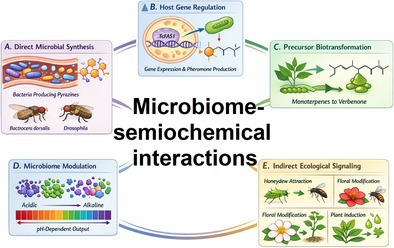

**ABBREVIATIONS**
AHL: N‐acyl homoserine lactone
BGCs: biosynthetic gene clusters
BVCs: bacterial volatile compounds
CHCs: cuticular hydrocarbons
CRISPR: clustered regularly interspaced short palindromic repeats
EAG: electroantennography
FISH: fluorescence *in situ* hybridization
GC–MS: gas chromatography–mass spectrometry
GC × GC: two‐dimensional gas chromatography
HS‐SPME: headspace solid‐phase microextraction
IPM: integrated pest management
MAGs: metagenome‐assembled genomes
MVOCs: microbial volatile organic compounds
mVOCs: microbial volatile organic compounds
NRPS: non‐ribosomal peptide synthetase
OR: odorant receptor
PKS: polyketide synthase
QS: quorum sensing
QSIs: quorum sensing inhibitors
RiPPs: ribosomally synthesized and post‐translationally modified peptides
RT‐qPCR: reverse transcription quantitative polymerase chain reaction
SIRM: stable‐isotope resolved metabolomics
SIT: sterile insect technique
SSR: single‐sensillum recording
VOCs: volatile organic compounds

## 1 INTRODUCTION

Chemical communication governs virtually all major behaviors of insects – mate finding, aggregation, foraging, and host selection – and underpins most semiochemical‐based pest management tools such as monitoring traps and mating disruption.^1^, ^2^, ^3^, ^4^ Traditionally, the focus has been on insect‐derived pheromones and plant volatiles as signal sources. However, an expanding body of work demonstrates that microbial partners – resident gut bacteria, yeasts, fungal endophytes and other symbionts – can produce, modify or mediate the semiochemicals that insects use to interact with their environment and with conspecifics.^5^, ^6^, ^7^ Microbe‐derived volatile organic compounds (mVOCs) include alcohols, acids, esters, sulfur compounds and nitrogenous volatiles; many are behaviorally active as attractants, repellents or modulators of insect olfactory systems.^8^, ^9^

Evidence for microbiome influence on insect chemical ecology has grown from three complementary lines of inquiry. First, ecological and analytical studies have repeatedly found that volatile blends which attract insects often contain fermentation‐derived compounds attributable to yeasts or bacteria.^10^, ^11^, ^12^ Second, manipulative microbiome experiments (axi‐/gnotobiotic rearing, antibiotics, recolonization with single strains) have demonstrated causal links between symbionts and semiochemical production or behavioral outcomes in several systems, notably German cockroaches and *Drosophila*.^13^, ^14^, ^15^ Third, genetic and biochemical studies reveal that symbionts can supply enzymes or precursors required for host pheromone biosynthesis or possess their own biosynthetic gene clusters that generate active volatiles.^6^

These findings have immediate translational relevance. Microbial volatiles have been exploited as lures: for example, fermentation‐based baits (yeast + sugar) substantially improve monitoring of the invasive fruit‐infesting *Drosophila suzukii*.^11^, ^16^ Conversely, bacterially produced deterrents such as fabclavines (from *Xenorhabdus* spp.) show promising repellent/deterrent activity against medically important mosquitoes, demonstrating that microbes are a reservoir for novel control chemistries.^17^ Beyond direct use of mVOCs, manipulating insect microbiomes (augmentation, paratransgenesis, or targeted disruption of key symbionts) offers conceptual routes to alter pest behavior or reduce pest fitness – tactics that could complement or extend classical integrated pest management (IPM) tools.^6^, ^18^, ^19^ Microbiome‐informed semiochemical tools can become robust, scalable additions to IPM.^20^, ^21^

Despite momentum, the field faces substantial limitations. Many published associations remain correlative: an insect and a volatile co‐occur, but the microbial source, biosynthetic pathway and behavioral target are not rigorously connected. Few studies combine metagenomics/metatranscriptomics, chemical analytics [gas chromatography–mass spectrometry/two‐dimensional gas chromatography (GC–MS/GC × GC)], functional genetics of microbes (knockouts/heterologous expression) and robust behavioral bioassays – the methodological pipeline required to move from observation to mechanism and application. Moreover, ecological contingencies (diet, developmental stage, plant microbiome, environmental factors) can change microbiome composition and volatile output, complicating generalization across taxa and contexts.^22^, ^23^

This review synthesizes the current state of microbiome–semiochemical research in insects with three objectives. First, we critically summarize mechanisms by which microbes shape semiochemical landscapes, namely direct VOC production, supply or modification of pheromone precursors, and alteration of host sensory physiology. Second, we evaluate behavioral outcomes across major insect orders (Diptera, Coleoptera, Hemiptera, Lepidoptera, Blattodea), emphasizing cases with causal or near‐causal evidence and discussing how microbial effects translate to aggregation, mating, host selection and disruption by pathogens. Third, we assess translational potential for pest management: (i) deploying microbial volatiles as attractants or repellents; (ii) engineering or augmenting microbiomes (including paratransgenesis); and (iii) disrupting symbiont pathways underpinning pest semiochemicals. Throughout we adopt a rigorous evidentiary standard and highlight experimental frameworks (multi‐omics, functional genetics and electrophysiology) required to validate causality and accelerate deployment in IPM.

By placing mechanistic understanding and pest management prospects at the center of the synthesis, we aim to provide a practical, rigorous roadmap for researchers and practitioners seeking to harness the microbiome–semiochemical axis for sustainable pest control.

## 2 MECHANISTIC FOUNDATIONS

**Figure 1:**
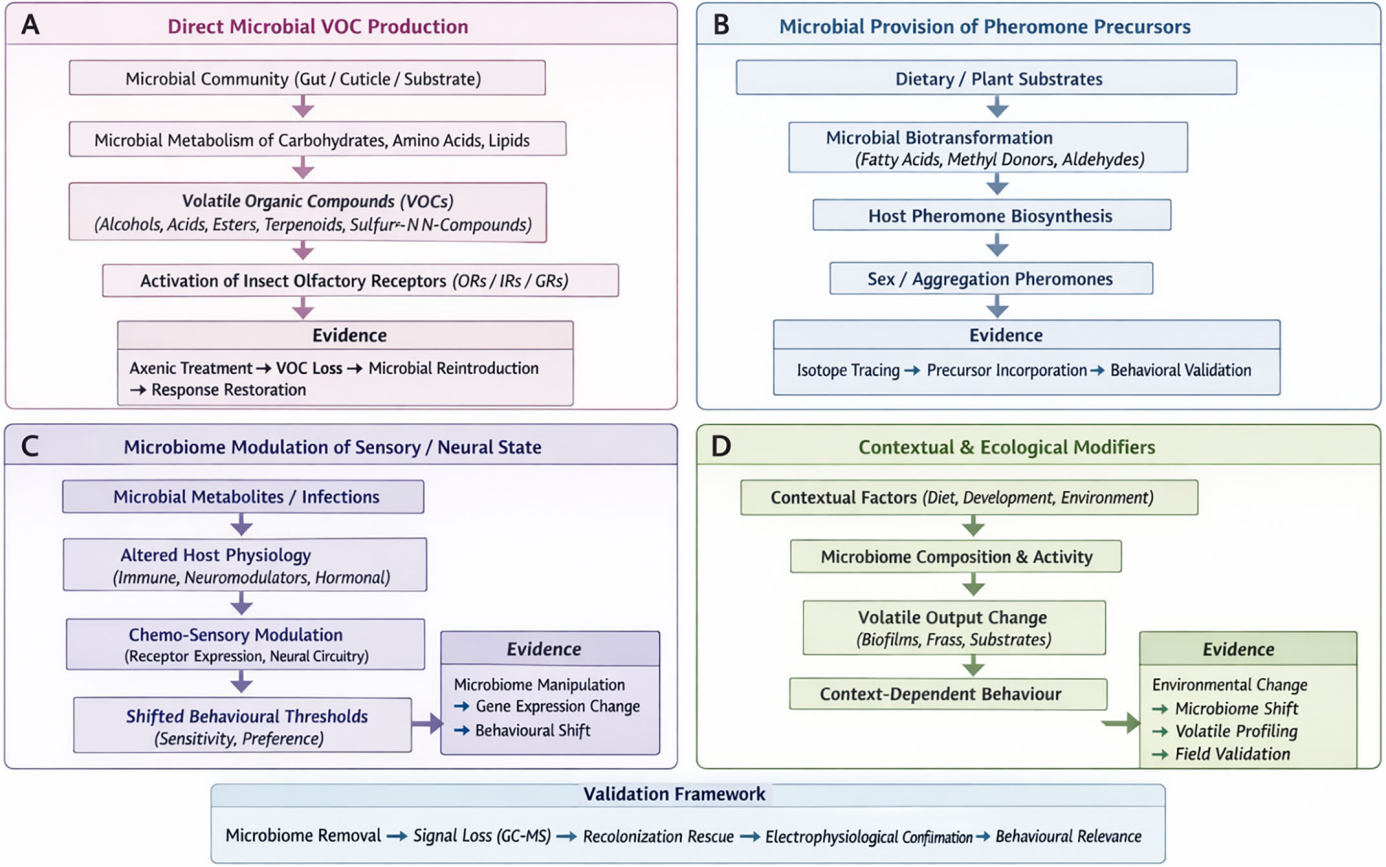
Mechanistic schematic. Microbiome → semiochemical → behavior pathways. Schematic illustrating the four principal mechanistic routes by which insect‐associated microbes influence semiochemical communication. (A) Direct microbial volatile organic compound (VOC) production. Gut, cuticular and substrate microbes synthesize VOCs (alcohols, acids, esters, terpenoids, sulfur‐ and N‐containing volatiles) that directly activate insect olfactory receptors and elicit attraction, repellence or oviposition responses. (B) Microbial provision/modification of pheromone precursors. Microbes transform dietary or plant substrates into small‐molecule precursors (e.g. short‐chain fatty acids, methyl donors) used by the host's biosynthetic machinery to synthesize sex or aggregation pheromones. (C) Microbiome modulation of host sensory/neural state. Microbial metabolites or infections alter the expression or sensitivity of odorant receptors and downstream neural circuits (gut–brain axis), modifying behavioral thresholds. (D) Contextual modifiers. Diet, developmental stage, quorum sensing and environmental microbiomes alter microbial composition and volatile output; biofilms on cuticle and frass act as persistent semiochemical sources. Insets indicate typical experimental evidence needed to validate each route [axenic treatment → gas chromatography–mass spectrometry (GC–MS) loss → recolonization rescue → electrophysiology/behavior].

Understanding how microbes alter insect chemical communication requires an explicit mapping from microbial genotype/metabolism → chemical product → host chemosensory detection → behavioral output. Empirical work to date supports four non‐exclusive mechanistic routes: (i) direct production of behaviorally active volatile organic compounds (VOCs) by microbes,^24^ (ii) microbial provision or modification of biochemical precursors used by the insect to synthesize pheromones or cuticular hydrocarbons (CHCs), (iii) microbiome regulation of host sensory and neural function, and (iv) context‐dependent modulation of the above by diet, ontogeny and ecological interactions.^7^ Figure 1 gives an overview of these four key mechanistic pathways. Below each route is synthesized with representative empirical examples, the mechanistic inferences that can be drawn, and the experimental approaches that give highest confidence of causality.

### 2.1 Direct production of semiochemicals by microbes

Microbial metabolism produces a chemically diverse volatilome (alcohols, acids, esters, ketones, pyrazines, sulfur‐ and nitrogen‐containing volatiles) that frequently overlaps with insect‐active semiochemicals.^8^, ^22^ Several lines of evidence support direct microbial production as an important source of behaviorally active cues.

First, chemical analyses of attractive odor blends often identify fermentation products traceable to yeasts or bacteria. For example, tephritid and drosophilid fruit flies are strongly attracted to mixtures dominated by ethanol, acetic acid, 3‐methyl‐1‐butanol and various esters – compounds produced by *Saccharomyces*, *Hanseniaspora* and various Enterobacteriaceae during fruit fermentation.^10^, ^11^, ^25^, ^26^ mVOCs, such as short‐chain alcohols and acids, act as attractive or repellent semiochemicals influencing insect feeding, aggregation, mating, and oviposition.^27^ Experimental baiting with live or fermenting cultures massively enhances trap captures relative to synthetic blends in several systems,^11^ indicating biological production rather than mere co‐occurrence is behaviorally relevant.

Second, manipulative microbiome work demonstrates microbial necessity or sufficiency for volatile production. In the German cockroach *Blattella germanica*, Wada‐Katsumata *et al*. showed that axenic (microbe‐free) individuals produced feces lacking multiple volatile carboxylic acids previously identified as aggregation pheromone components; recolonization with gut bacteria restored these VOCs and aggregation behavior.^14^ Direct experimental evidence from microbiome removal and reintroduction demonstrates that microorganisms themselves synthesize insect‐active VOCs that mediate behavioral responses, such as aggregation and host location.^28^ This experimental chain – removal, chemical absence, behavioral loss, and rescue – is the strongest evidence for direct microbial production.

Third, targeted screens of microbial cultures have yielded novel bioactive metabolites with repellent or deterrent activity. Entomopathogenic bacteria of the genus *Xenorhabdus* produce fabclavines and related secondary metabolites that strongly deter mosquito biting and oviposition; extracts and purified compounds reduce feeding more effectively than conventional repellents in laboratory assays.^17^ Similarly, *Streptomyces* and other soil microbes synthesize geosmin and 2‐methylisoborneol, which are detected by insect olfactory circuits as indicators of harmful microbes and often elicit aversion.^29^, ^30^ Similarly, cereal leaf beetle‐associated bacteria increase both the number and emission of wheat volatiles, including hydrocarbons, benzenoids, esters, and lactones, especially during larval feeding, amplifying plant chemical defenses that can influence insect behavior.^31^

Finally, phylogenomic and metabolomic surveys show conserved biosynthetic genes in microbes that predict production of insect‐active volatiles.^6^ Discovery pipelines that combine culture‐dependent volatile profiling (headspace GC–MS/GC × GC) with metagenomics accelerate identification of candidate mVOCs and their genetic bases.^22^ Together, these approaches demonstrate that microbial biosynthetic capacity frequently explains otherwise puzzling semiochemical emissions in insect ecologies.

#### 2.1.1 Evidence strength and recommended assays

The strongest case for direct microbial production uses: (i) removal of microbes (axenic or antibiotic treatments), (ii) chemical profiling showing loss of compound(s), (iii) behavioral loss, and (iv) reintroduction/single‐strain colonization or heterologous expression that restores both compound and behavior.^14^, ^32^ Complementary evidence includes volatile production by pure cultures and gene knockouts in the microbe that eliminate production of the candidate compound.

### 2.2 Microbe‐mediated modulation of host chemical synthesis

Microorganisms can supply precursors, enzymes, or cofactors required for insect biosynthesis of semiochemicals – a route that renders the microbe essential to host pheromone chemistry even when the final product is synthesized by host enzymes.^6^, ^33^ Mechanisms include microbial transformation of dietary substrates into pheromone precursors, microbial provision of short‐chain fatty acids or methyl donors for hydrocarbon modifications, and microbial expression of biosynthetic enzymes active in host tissues.

#### 2.2.1 Representative examples include

1. Bark beetles and gut/wood microbes. Several studies indicate that gut and symbiotic microbes in wood‐boring beetles transform host tree compounds (monoterpenes and phenolics) into aggregation pheromone components such as verbenone and guaiacol; antibiotic treatments or elimination of microbial partners reduces pheromone yield in some systems.^6^ Although the exact microbial genes vary by system, the biochemical logic often involves oxidation, demethylation or decarboxylation of plant‐derived substrates.
2. CHCs in social insects and *Drosophila*. In termites and some ants, gut microbes provide methyl‐branched chain precursors (e.g. propionate, methylmalonate) used by the host to produce species‐ and colony‐specific cuticular hydrocarbons.^6^, ^34^ In *Drosophila*, early high‐profile work reported that commensal *Lactobacillus* spp. were associated with changes in CHC profiles and assortative mating,^13^ suggesting microbes can alter host pheromone biosynthesis; although reproducibility across laboratories has been variable, more recent studies using gnotobiotic approaches confirm microbe‐linked modulation of CHC composition in some contexts.^15^
3. Provision of enzymatic activity. Some microbes express enzymes (e.g. alcohol dehydrogenases, decarboxylases, acetyltransferases) that convert host or dietary metabolites into pheromonal moieties that the insect then packages or modifies further. Experimental validation comes from demonstrations that removing the microbe leads to loss of a pheromone while expressing the microbe's enzyme heterologously restores production (case reports remain few, representing a priority experimental gap; e.g. Ren *et al*. showing *Bacillus* rectal bacteria directly produce sex pheromone pyrazines in *Bactrocera dorsalis* and gut bacteria‐mediated aggregation pheromone release in *Trigonorhinus* sp.).^35^, ^36^

#### 2.2.2 Evidence strength and experimental needs

Demonstrating microbial contribution to host biosynthesis requires tracing metabolic flux: isotope (^13^C/^2^H) labeling of dietary precursors combined with metatranscriptomics and host/microbe‐specific enzyme assays can show whether carbon flows through microbial enzymes into host pheromones. Genetic disruption (microbial gene knockouts or silencing) that blocks host pheromone production provides the gold standard, but such tools are currently available for only a handful of culturable symbionts.^37^

### 2.3 Microbiota influence on host sensory and neural systems

A growing literature shows that microbial status can alter insect chemosensory perception and downstream neural processing; that is, microbes change not the signal, but the receiver. Mechanisms include microbial modulation of receptor expression, neuromodulator production that alters neuronal excitability, and influence on development of olfactory circuits.

#### 2.3.1 Key findings

1. Olfactory preference shifts. Manipulating the gut microbiota of *Drosophila* (axenic rearing, antibiotic treatment, or recolonization) changes adult preference for microbe‐derived odors and nutritional cues.^15^, ^38^ These behavioral changes are accompanied by altered expression of gustatory and olfactory genes in some studies, suggesting transcriptional reprogramming of sensory pathways.^15^
2. Dedicated microbial odor circuits. Flies possess specialized circuits for microbe‐indicative volatiles. For geosmin – an odorant produced by soil microbes – *Drosophila* evolved a narrowly tuned olfactory receptor (Or56a) and an antennal lobe circuit promoting avoidance, linking a microbial product to a dedicated neural pathway and behavioral outcome.^29^ This example shows that insects can evolve sensory specializations for microbial cues and that microbes can thereby drive sensory ecology.
3. Neuroactive microbial metabolites. Microbes produce neuroactive small molecules (short‐chain fatty acids, gamma Aminobutyric acid (GABA), indoles) with potential to modulate host neurophysiology. Although the gut–brain axis is better characterized in vertebrates, insect examples suggest microbial metabolites can affect locomotion, learning and foraging drive.^15^, ^39^, ^40^ For instance, parasite infection reduces sucrose and odor responsiveness in bumble bees, plausibly via immune‐ or metabolite‐mediated effects on neural circuits.^39^

#### 2.3.2 Evidence strength and assays

Linking microbes to sensory/neural modulation requires combining microbiome manipulations with electrophysiology (electroantennography, single‐sensillum recording), transcriptomics of sensory tissue, and behavioral assays. Demonstrating altered receptor binding or neuronal firing in response to microbe‐dependent volatiles, ideally alongside rescue experiments, is the most persuasive evidence.

### 2.4 Contextual modifiers: diet, development, community and environment

All the above mechanisms are strongly modulated by context: diet composition, developmental stage, host genotype, co‐occurring microbes (microbial community interactions) and the surrounding environment (plant microbiome, abiotic factors) frequently change volatile output and insect responses.

#### 2.4.1 Diet/matrix effects

The substrate on which microbes grow radically alters their volatilome (sugar *versus* fruit pulp *versus* plant tissue). For example, *Saccharomyces* and Hanseniaspora produce very different volatile profiles when fermenting sucrose *versus* fruit substrates, shifting attractant potency for *Drosophila* fruit flies.^11^ Thus, field lure performance can differ from laboratory results if culture media are not ecologically relevant.

#### 2.4.2 Developmental and seasonal shifts

Microbiome composition changes across larval to adult stages and seasonally, with corresponding changes in semiochemical profiles.^15^ Insects that undergo metamorphosis may acquire distinct adult‐stage symbionts that alter adult pheromone economics.

#### 2.4.3 Microbial interactions and horizontal acquisition

Symbiont composition is often horizontally acquired from diet and environment; thus, plant or fruit microbiomes can determine which symbionts colonize insects and what volatiles are produced.^41^ Plant pathogens that alter plant VOCs can indirectly change vector behavior, as seen with *Candidatus Liberibacter* in citrus increasing methyl salicylate and attracting psyllid vectors.^42^ Microbial plant pathogens can also modify host‐plant volatile blends to attract vector insects; such pathogen‐induced odor shifts may function as allomones and could be exploited in lure‐ or trap‐based monitoring systems.^43^, ^44^ Plant microbiome can determine which symbionts colonize insects and what volatiles are produced.^41^

#### 2.4.4 Implications for inference and application

These contextual dependencies mean that mechanistic claims must be validated under ecologically realistic conditions (field trials, relevant substrates, natural diets) before translational deployment. For monitoring or attractant development, lure optimization must consider substrate composition, microbial growth phase, and environmental odor background.

#### 2.4.5 Synthesis and methodological checklist

**Figure 2:**
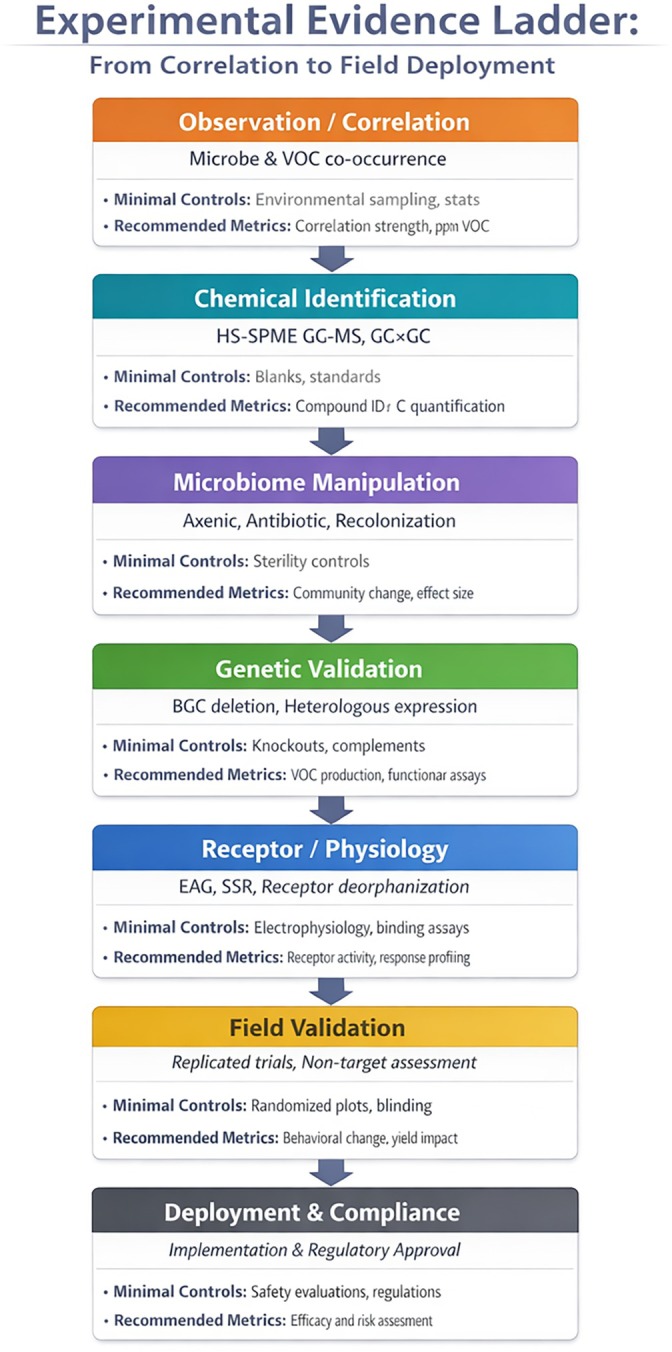
Experimental evidence ladder/decision tree. From correlation to field deployment. A vertical ladder/flowchart showing the recommended pipeline for establishing mechanistic and translational claims. (1) Observation/correlation [co‐occurrence of microbe and volatile organic compound (VOC)]. (2) Chemical identification [headspace solid phase microextraction (HS‐SPME0, gas chromatography–mass spectrometry (GC–MS), two‐dimensional gas chromatography (GC × GC)). (3) Microbiome manipulation (axenic, antibiotic, recolonization). (4) Genetic validation [microbial biosynthetic gene cluster (BGC) deletion or heterologous expression]. (5) Receptor/physiology [electroantennography/single‐sensillum recording (EAG/SSR), receptor deorphanization]. (6) Field validation (replicated trials, non‐target assessment). (7) Deployment and regulatory compliance. Each step lists minimal experimental controls and recommended outcome metrics (e.g. parts per million VOC, trial replication, effect size thresholds).

To rigorously map microbe → semiochemical → behavior pathways researchers should combine: (i) high‐resolution chemical profiling [headspace solid‐phase microextraction (HS‐SPME) GC–MS, GC × GC–MS]; (ii) controlled microbiome manipulations (axenic hosts, antibiotic treatments with recolonization, gnotobiotic rearing); (iii) microbial genomics/transcriptomics to identify candidate biosynthetic genes; (iv) functional genetics (microbial knockouts or heterologous expression); and (v) electrophysiology/behavioral bioassays [electroantennography (EAG), single‐sensillum recording (SSR), choice tests, field trials]. Use of stable isotope tracing (^13^C labeling) is especially powerful for proving carbon flux from microbial metabolism into host pheromones. Adoption of these multi‐layered pipelines will convert many correlative reports into mechanistically validated systems suitable for translational development. Figure 2 illustrates this progression from correlation to validated deployment as an evidence ladder, with key steps, controls, and metrics.

### 2.5 Quorum sensing and regulation of volatile output

Quorum sensing (QS) – population density‐dependent, small‐molecule signaling [*N*‐acyl homoserine lactones (AHLs) in Gram‐negatives; oligopeptides in Gram‐positives; AI‐2 interspecies signals] – coordinates many collective microbial behaviors (biofilm formation, secondary metabolism, virulence) and is increasingly recognized as a regulator of VOC production.^45^, ^46^, ^47^ QS circuits can upregulate or downregulate enzymes and pathways that produce terpenoids, sulfur/nitrogen volatiles and other bacterial volatile compounds (BVCs) that interact with insects.^46^, ^48^, ^49^

#### 2.5.1 Concrete examples and relevance

1. QS‐dependent VOC expression. In several plant‐associated bacteria (e.g. some *Pseudomonas*, *Enterobacter* spp.), disruption of AHL‐based QS lowers emission of VOCs (alcohols, ketones, hydrocarbons) that influence plant–insect interactions; conversely, QS activation increases VOC output.^46^, ^50^ This implies that microbial population density (and therefore substrate/host state) can determine whether a microbe produces an attractant or repellant.
2. Ecological consequences. For insects that cue on mVOCs to find food or oviposition sites, QS‐regulated switches can produce threshold effects – a once‐sparse microbial community produces negligible cue, but beyond a density threshold the same microbe becomes a strong attractant. This explains why aging or fermenting fruits (high microbial biomass) are more attractive than freshly colonized substrates.^11^, ^46^
3. Mechanistic tests. Demonstrating QS control of insect‐active volatiles uses QS mutants (e.g. luxI/luxR or easI/easR knockouts), chemical QS inhibitors (QSIs) or exogenous addition of autoinducers, combined with volatile profiling (HS‐SPME GC–MS) and behavioral bioassays (choice tests, trap catches). De Clerck *et al*. review approaches and provide examples where QS mutant strains produce distinctly altered VOC bouquets and altered attractiveness to insects.^46^
4. Implication for pest control. QS is a potential manipulation point. QS inhibitors or manipulation of microbial density could suppress the production of attractant VOCs in the field (reduce ‘pull’ to crops), or conversely, QS‐induced microbe augmentations could be used to amplify baits for traps.

### 2.6 Microbial biofilms and cuticular/surface microbiomes as semiochemical sources

Most studies focus on gut microbes, but microbes living on external surfaces (cuticle, oviposition substrate, frass) and biofilms adhered to cuticle or egg masses also produce volatiles or carry odorant‐active exudates that influence insect communication and interspecific interactions.^51^ The cuticular microbiome can be stable and produce semiochemicals that are effectively part of the insect's ‘extended phenotype’.

#### 2.6.1 Key points and evidence

1. Surface‐associated microbes produce semiochemicals. Microbial communities on insect cuticle or in feces (e.g. cockroach feces) synthesize volatile carboxylic acids and other aggregation cues.^14^ In social insects, surface microbes can contribute to nest odor profiles and affect nestmate recognition or pathogen avoidance.^51^, ^52^
2. Biofilms as sustained release sources. Biofilms persist on surfaces and can continuously emit mVOCs, acting as slow‐release semiochemical sources. For oviposition sites or traps, biofilms can therefore maintain attractive plumes longer than transient planktonic cultures.^22^, ^53^
3. Experimental approach. Distinguish gut *versus* cuticular sources by sequentially sampling and profiling volatiles from: (i) insect surface washes/swabs (biofilm extracts), (ii) feces/frass headspace, and (iii) gut homogenates. Combine culture‐dependent and culture‐independent (amplicon/shotgun metagenomics) approaches to identify candidate taxa, then test pure cultures or biofilm mimics in behavioral assays. Studies that suppressed or altered surface microbiomes (e.g. topical antimicrobials) and recorded changes in cuticular hydrocarbons or odor emission provide the most direct evidence.^54^
4. Implication for pest control. Surface microbial communities could be manipulated (topical probiotics or microbial antagonists) to change pest semiochemical signatures (reduce aggregation or mating signals on crop surfaces) or to render traps more persistent by engineering biofilms that emit attractive VOCs. Recent studies illustrate applied strategies: (i) microbe‐derived volatiles from aphid honeydew can recruit ladybird beetles to enhance biological control of aphids (4‐isopropylbenzyl alcohol as a key cue); and (ii) engineering insect–microbe symbioses may allow disruption of pest signaling or increased susceptibility to biocontrol agents, highlighting the potential of microbiome‐based interventions for sustainable, eco‐friendly pest management.^55^, ^56^, ^57^

### 2.7 Biosynthetic gene clusters and specific gene families to emphasize

To move beyond correlation, we must link candidate volatile molecules to microbial genetic machinery. Genome mining and BGC analysis (antiSMASH, MIBiG) provide powerful ways to identify candidate pathways in symbiont genomes/metagenomes.^58^, ^59^ Several BGC families are particularly important for insect‐active metabolites:

1. Terpene synthase and terpene BGCs. Many microbial volatiles that mediate insect behavior are terpenoids (e.g. geosmin is a sesquiterpene‐related compound produced by *Streptomyces* and other bacteria). Terpene synthase genes in bacteria and fungi predict terpene VOC production; these BGCs often include prenyltransferases and tailoring enzymes (oxidases, methyltransferases).^60^ antiSMASH identifies terpene clusters and can be used to compare to known terpene producers in MIBiG.
2. Polyketide synthase (PKS) and non‐ribosomal peptide synthetase (NRPS) clusters. PKS/NRPS clusters underpin diverse secondary metabolites (some volatile or semi‐volatile after tailoring) with strong bioactivity (e.g. insecticidal or deterrent activity). *Xenorhabdus* fabclavines (putatively NRPS/PKS hybrid products) are an example of microbially produced feeding‐deterrents with insect behavioral effects.^19^ Genome mining can pinpoint candidate PKS/NRPS clusters responsible for these compounds.
3. Ribosomally synthesized and post‐translationally modified peptides (RiPPs) and small volatile RiPP‐derived derivatives. RiPPs and their tailoring enzymes can yield small volatile derivatives or precursors that affect insect behavior. antiSMASH and MIBiG annotate RiPP classes and provide templates for heterologous expression.
4. Tailoring enzymes and small‐molecule‐producing operons. Single enzymes (alcohol dehydrogenases, decarboxylases, methyltransferases, hydrolases) often determine whether a pathway yields an odorant molecule (e.g. production of acetoin/diacetyl from pyruvate via acetolactate synthase/acetolactate decarboxylase). Identifying these genes in symbiont genomes (and confirming their expression by metatranscriptomics) is often the most direct route toward assigning function. antiSMASH and comparative genomics followed by heterologous expression provide a strong proof path.^58^, ^59^

### 2.8 Experimental pipeline to connect BGC → VOC → behavior

1. Genome/metagenome mining. Identify candidate BGCs in cultured symbiont genomes or metagenome‐assembled genomes (MAGs) with antiSMASH and compare with characterized BGCs in MIBiG (e.g. identification of hundreds of biosynthetic gene clusters from insect‐associated symbionts^61^).
2. Expression evidence. Use metatranscriptomics/quantitative polymerase chain reaction with reverse transcription (RT–qPCR) to show candidate genes are expressed *in situ* (e.g. in gut or on cuticle) at times when the VOC is produced.
3. Functional validation. Delete or silence (CRISPR, allelic exchange, transposon mutagenesis) key biosynthetic genes and show loss of the VOC in culture and in the host; or express the cluster/genes heterologously in a model microbe (e.g. *Escherichia coli* or *Streptomyces*) and show gain of VOC production.
4. Behavioral proof. Demonstrate that the VOC(s) lost in the mutant are behaviorally active (EAG/SSR + behavioral choice assays + field validation).

This BGC‐centric approach is the route to mechanistic rigor: it converts correlative VOC profiles into gene‐to‐behavior chains that can be targeted or exploited for pest control.

## 3 BEHAVIORAL OUTCOMES

Microbiome–semiochemical interactions translate into changes in insect behavior across multiple functional classes relevant to pest management,^19^ including aggregation and social behavior, mate recognition and reproductive behavior, host location and feeding, and behavioral disruption mediated by pathogens or parasites. Across these behavioral domains, we synthesize mechanistic links connecting microbes, semiochemicals, and insect sensory or behavioral responses, highlight representative and rigorously studied examples spanning multiple insect orders, and critically evaluate the strength of evidence using an evidence‐gradient framework ranging from correlative observations to manipulative and genetically supported studies, together with methodological considerations relevant to both researchers and IPM practitioners.

Table 1 summarizes high‐confidence systems supported by manipulative, genetic, or field‐validated evidence, whereas Table 2 compiles emerging or predominantly correlative systems that remain mechanistically unresolved or insufficiently validated under operational conditions. Together, these tables operationalize the evidence‐gradient framework by explicitly distinguishing systems with established causal inference from those requiring further mechanistic and field‐level validation. Both tables additionally identify key experimental gaps limiting causal inference or translational implementation, thereby highlighting priority directions for future research and application in IPM.

**Table 1:** High‐confidence microbe‐mediated semiochemical systems supported by manipulative, genetic, or field‐validated evidence

No.	Pest/system	Microbial partner	Semiochemical(s) implicated	Behavioral outcome	Evidence category	Key experimental gap	References
1	German cockroach, *Blattella germanica*	Gut bacterial community	Short‐chain carboxylic acids and fecal volatiles	Conspecific aggregation	Manipulative	Lack of operational validation under complex urban infestation conditions	15
2	Spotted‐wing drosophila, *Drosophila suzukii*	Fermentation‐associated yeasts and bacteria (*Saccharomyces*, *Hanseniaspora*, Enterobacteriaceae)	Ethanol, acetic acid, fusel alcohols, esters	Attraction to oviposition and feeding sites	Manipulative + Field‐validated	Need standardized bait formulations, longevity optimization, and climatic robustness testing	10, 11
3	Mexican fruit fly, *Anastrepha ludens* and related tephritids	Enterobacteriaceae (*Klebsiella*, *Enterobacter*)	3‐Methyl‐1‐butanol, ammonia, bacterial VOCs	Attraction to host substrates and traps	Manipulative + Field‐validated	Limited comparative validation across tephritid species and agroecosystems	11, 25, 62
4	*Drosophila paulistorum*	*Wolbachia*	Altered CHC and pheromone blends	Reduced mating success following symbiont disruption	Genetic + Manipulative	Limited ecological validation outside laboratory populations	63
5	Mosquito vector control systems	*Xenorhabdus* spp.	Fabclavines and related secondary metabolites	Feeding deterrence and repellency	Manipulative	Absence of field deployment, toxicological scaling, and formulation studies	17
6	Asian citrus psyllid, *Diaphorina citri*	*Candidatus Liberibacter*–mediated alteration of plant microbiome/physiology	Methyl salicylate and related plant volatiles	Enhanced vector attraction to infected hosts	Manipulative + Field‐validated	Mechanistic separation of direct pathogen effects from broader microbiome‐mediated changes	42

Abbreviations: CHC, cuticular hydrocarbon; VOC, volatile organic compound.

**Table 2:** Emerging or partially validated microbe‐mediated semiochemical systems requiring further mechanistic or translational resolution

No.	Pest/system	Microbial partner	Semiochemical(s) implicated	Behavioral outcome	Evidence category	Key experimental gap	References
1	*Drosophila melanogaster*	Commensal bacteria (*Lactobacillus* spp.)	Altered CHC profiles and volatile blends	Assortative mating and mate preference	Manipulative (limited reproducibility evidence)	Inconsistent reproducibility and incomplete mechanistic resolution of microbiome effects on CHCs	13, 15
2	Bark beetles/wood‐borers (*Dendroctonus* spp.)	Gut‐ and gallery‐associated bacteria and fungi	Verbenone, verbenol, guaiacol, terpene derivatives	Aggregation and anti‐aggregation signaling	Correlative + Partial manipulation	Lack of microbial gene‐level validation and insufficient microbiome‐resolution studies under field conditions	64
3	Mosquitoes (*Aedes*, *Anopheles*) – host attraction	Human skin microbiota (*Staphylococcus*, *Corynebacterium*)	Carboxylic acids, aldehydes, skin VOCs	Differential host attractiveness	Correlative + Manipulative	Need longitudinal and controlled microbiome engineering studies to establish durable causal relationships	65, 66
4	Termites (*Zootermopsis nevadensis*)	Gut microorganisms	Precursors involved in CHC biosynthesis	Colony and nestmate recognition	Manipulative (biochemical tracing)	Absence of direct microbial genetic validation and colony‐level ecological confirmation	34
5	Stored‐grain pest systems	Grain‐associated fungi and bacteria	Aldehydes, alcohols, ketones, acids	Attraction to conditioned grain	Correlative + Laboratory validation	Lack of operational‐scale validation and standardized microbial characterization across storage environments	22, 31

Abbreviations: CHC, cuticular hydrocarbon; VOC, volatile organic compound.

### 3.1 Aggregation and social behavior

Aggregation pheromones concentrate conspecifics at feeding or sheltering sites and often derive from feces, frass or substrate fermentation – matrices that are microbially rich. Several rigorous studies provide near‐causal chains linking microbes to aggregation semiochemicals.

A canonical example is the German cockroach *Blattella germanica*. Wada‐Katsumata *et al*. showed that axenic cockroaches (devoid of gut microbes) produced feces lacking many volatile carboxylic acids previously identified as components of the aggregation pheromone; recolonization with gut bacteria restored both the volatile profile and aggregation behavior.^14^ This study followed a strong evidentiary pipeline: microbiome removal → chemical loss (GC–MS) → behavioral loss (choice assays) → microbial rescue (recolonization), and therefore provides direct proof that gut (and fecal) microbes produce behaviorally active aggregation cues.^14^

In wood‐boring and bark beetles, microbial transformation of host tree compounds often produces aggregation or anti‐aggregation signals. Classic and modern work shows that bacterial and fungal symbionts convert monoterpenes and phenolics to pheromonal components (e.g. verbenone, verbenol, guaiacol) that modulate mass‐attack dynamics.^64^, ^67^, ^68^ In many bark beetle systems, evidence ranges from culture‐based volatile identification and field attraction trials to manipulative work that suppresses symbionts and observes pheromone changes – that is, a mixture of correlative and manipulative evidence that nonetheless points to essential microbial roles in mass‐attack signaling.^6^, ^69^

Social insects (termites, ants) also show microbiome contributions to nest odor and colony recognition. Termite gut microbes contribute precursors for methyl‐branched hydrocarbons used in CHC signatures,^34^ and experimental manipulations of surface microbiomes can alter nest odors and social recognition in a few ant and wasp systems.^34^, ^70^, ^71^ Across aggregation cases, the strongest evidence shares the Wada‐Katsumata pattern: controlled microbiome manipulation, paired chemical analytics, and behavioral assays.^14^, ^34^ Teseo *et al*. showed that experimentally altering gut microbes in *Acromyrmex echinatior* ants changed aggression and cuticular chemical profiles in nestmate interactions, implying microbial influence on nestmate recognition.^72^

#### 3.1.1 Methodological note (aggregation)

For convincing claims, authors should present: (i) headspace profiling of feces/frass/host substrate; (ii) manipulation of microbiome (axenic/antibiotic, topical antimicrobials, or selective recolonization); and (iii) behavior tested using both laboratory choice assays and – where feasible – field trap trials to confirm ecological relevance.

### 3.2 Mate recognition and reproductive behaviors

Microbial effects on mating arise through at least two routes: microbes altering the chemical signature of sexual signals (e.g. CHCs, sex pheromones) and microbes modulating the sensory or physiological state of potential mates.

Early, high‐impact work in *Drosophila melanogaster* showed that diet‐driven changes in gut microbiota correlate with assortative mating: flies reared on different diets developed mating preferences for same‐diet mates, and antibiotic treatment removed this preference.^13^ Sharon *et al*. reported changes in CHC profiles associated with particular bacteria (*Lactobacillus*), proposing a causal microbial role in sexual signaling. The report stimulated extensive follow‐up and debate about reproducibility across labs and genotypes; subsequent work by Wong *et al*. refined the picture by showing that microbiota identity can shape olfactory preferences and foraging decisions, thereby indirectly affecting mating contexts. Thus, in *Drosophila* the evidence is mixed but increasingly points to microbiome‐linked modulation of sexual communication under particular dietary and genetic contexts.^13^, ^15^

A strong, mechanistic example of symbiont‐driven mating outcomes is *Drosophila paulistorum* infected with *Wolbachia*: Schneider *et al*. demonstrated that *Wolbachia* removal changed male CHC profiles, led to absence of *Wolbachia* in pheromone‐producing cells, and caused males to be rejected by wild females – establishing a direct link between symbiont presence, pheromone chemistry, and mating success.^63^ This constitutes near‐gold standard evidence because it couples symbiont perturbation, chemical phenotype, localization of symbionts in pheromone tissues, and mating behavior.

Other insect taxa show suggestive but less complete evidence. Termite and ant CHCs may be partially microbiome‐derived; experimental topical manipulations sometimes alter recognition or mating cues, but the causal gene‐to‐molecule pathways remain to be validated in most cases.^34^, ^51^ Overall, mate choice effects exist in diverse taxa, but the most convincing systems (*Wolbachia*/*D. paulistorum*, cockroach aggregation) are those that couple symbiont perturbation with direct chemical and behavioral readouts.

#### 3.2.1 Methodological note (mating)

To be persuasive, demonstrate: (i) symbiont localization in pheromone‐producing tissues (histology/fluorescence *in situ* hybridization); (ii) chemical profiling of pheromone blends (GC–MS) with quantification; and (iii) mating assays that include antibiotic controls, recolonization, and – where possible – knockouts of microbial biosynthetic genes.

### 3.3 Host location and feeding behavior

Many foraging and oviposition decisions are guided by microbial VOCs from food/host substrates, insect frass, or host‐associated microbes; here the evidence base is large and translationally promising.

#### 3.3.1 Fruit flies and fermenting microbes

Tephritids and drosophilids are archetypal examples: yeast and enteric bacteria fermenting fruit produce ethanol, acetic acid, fusel alcohols and esters that strongly attract adult flies.^10^, ^25^, ^62^ Traps baited with fermenting yeast/sugar lures consistently outperform vinegar or synthetic lures for *D. suzukii* and other fruit flies in field trials,^11^ providing applied proof that microbial volatiles drive attraction and can be harnessed for monitoring and control.^11^

#### 3.3.2 Tephritid larval gut microbes and host suitability

In several fruit‐infesting flies, larval gut bacteria facilitate digestion of fruit substrates and produce volatiles that both attract adults and condition oviposition sites; manipulative experiments (antibiotics, recolonization) show effects on larval survival and female preference in some species, linking microbial physiology to host range and pest potential.^10^, ^73^, ^74^

#### 3.3.3 Mosquito host attraction and skin microbiota

Human–vector interactions illustrate microbiome‐mediated host selection: variation in human skin microbiota composition predicts differential attractiveness to *Anopheles* and *Aedes* mosquitoes because skin bacteria produce carboxylic acids and other volatiles that mosquitoes use as cues.^65^, ^66^, ^75^ Recent studies identify specific bacterial taxa (*Staphylococcus*, *Corynebacterium*) and their volatiles as drivers of mosquito preference, and even show proof‐of‐concept for altering attractiveness by changing skin microbiome composition.^65^, ^66^, ^75^ This body of work underscores microbiome manipulation as a potential vector control strategy (microbiome engineering of hosts or baits).

#### 3.3.4 Strength of evidence (host location)

For fruit flies and mosquitoes, the evidence is strong: chemical identification of active mVOCs, laboratory and field attraction assays, and microbiome manipulations (culture baits, antibiotic treatments, *in vitro* microbial communities) demonstrate a causal role. For other taxa (many Lepidoptera, Hemiptera), evidence is emerging but often more correlative (volatile profiling without manipulative recolonization).

#### 3.3.5 Methodological note (host location)

Because substrate (fruit type, decay stage) and microbial growth phase strongly affect VOC bouquets, authors should explicitly test lure formulations across realistic substrates and time scales and use field trials to confirm laboratory findings.

### 3.4 Disruption by pathogens and parasites

Pathogen or parasite infection can alter host semiochemical landscapes and behavioral responsiveness, sometimes indirectly through immune‐mediated metabolic shifts or direct microbial metabolism of host molecules.

#### 3.4.1 Bee pollinators and gut parasites

Infection by the trypanosomatid *Crithidia bombi* in bumblebees affects foraging behavior, sucrose responsiveness, and cognitive performance.^76^, ^77^ Microbiome structure can modulate susceptibility to *Crithidia* and thus indirectly influence foraging and pollination behavior.^77^ These studies show parasites can change both the production and perception of feeding cues, with consequences for colony fitness and pollination services.

#### 3.4.2 Virus and bacterial infections in social insects

Viral infection in honey bees alters odor responsiveness and learning, and some pathogens manipulate plant volatiles to increase vector attraction, although mechanistic details linking pathogen metabolism to host semiochemical disruption vary among systems.^42^, ^78^

#### 3.4.3 Implication

Pathogens can reduce the reliability of semiochemical cues or change host attractiveness; for pest management this is double‐edged – pathogen‐induced changes may reduce pest fitness (beneficial) but can also compromise semiochemical monitoring if traps rely on signals altered by infection.

#### 3.4.4 Methodological note (pathogen disruption)

Integrative studies combining infection assays, metabolomics of host volatiles, EAG, and behavior are required to dissect pathogen effects on signal production *versus* perception.

## 4 MICROBIOME‐MEDIATED SEMIOCHEMICALS AS PEST CONTROL TOOLS

Microbe‐derived semiochemicals and the microbes that produce them present multiple actionable avenues for pest management. Below we synthesize current approaches, evaluate their evidence base and feasibility, and propose experimental and deployment frameworks that emphasize robustness, biosafety and integration with IPM.

### 4.1 Exploiting microbially produced VOCs as lures and repellents

The simplest translational route is to deploy mVOCs directly as attractants or repellents. Microbe‐derived semiochemicals present multiple actionable avenues for pest management.^54^ Multiple pest systems already use fermentation‐based lures: for tephritids and drosophilids, traps baited with fermenting yeast or bacterial cultures (or their headspace blends) outperform standard vinegar lures in both laboratory and field trials.^10^, ^11^, ^25^, ^62^ These lures exploit robustly produced compounds (ethanol, acetic acid, fusel alcohols, esters) and are attractive because microbial metabolism generates dynamic plumes that mimic real oviposition or feeding sites,^11^, ^79^ and emerging work shows that bacterial VOCs can alter insect behavior and even mortality in stored‐product pests like *Sitophilus zeamais*.^80^

On the repellent side, microbial secondary metabolites (e.g. fabclavines from *Xenorhabdus*) show potent deterrent activity against mosquitoes,^17^ and microbially produced volatile geosmin elicits aversive behavior in *Drosophila* and other insects.^29^, ^60^ These findings indicate mVOCs can substitute for or augment synthetic repellents; because microbial products frequently evolve to target specific ecological responses, they can offer novel modes‐of‐action distinct from existing chemistries.^17^, ^79^

Key developmental needs for deployable mVOCs include: (i) identification and standardization – chemically identify active constituents using HS‐SPME GC–MS/GC × GC coupled to behavioral bioassays and EAG/SSR to pinpoint receptor‐active compounds^8^, ^22^; (ii) formulation and release – develop stable dispensers or culture‐based dispensers (live fermentations, slow‐release gels) that mimic the temporal dynamics of natural microbial plumes – field tests must evaluate longevity and non‐target attraction; and (iii) optimization for background odor – assess lure performance against site‐specific odor backgrounds (crop volatiles, ambient microbial odor) to avoid saturation or interference.^11^

### 4.2 Microbiome engineering and paratransgenesis: conceptual and practical pathways

A more ambitious set of tools aims to engineer microbes – either those associated with pests or those applied to crops/traps – to produce desired semiochemicals or disrupt pest communication. Two overlapping approaches are relevant: first, engineering free‐living microbes (or probiotics) used as baits/trap enhancers; and second, paratransgenesis – genetically modifying insect symbionts to express effector molecules that change host behavior or fitness.

1. Engineered baits/probiotics. Yeast or bacterial strains can be selected or modified to overproduce attractive volatiles (e.g. acetoin, acetaldehyde, particular esters) or repellents. Successful laboratory modification of acetic acid bacteria to upregulate acetoin production increased *Drosophila* attraction.^10^, ^81^ Engineered bait microbes can be applied to trap substrates or ‘attract‐and‐kill’ stations; their advantage is renewable volatile production and potential cost‐effectiveness. However, formulation control, regulatory approval, and horizontal transfer risk must be addressed.
2. Paratransgenesis and *in situ* symbiont editing. Paratransgenesis – the modification of insect symbionts to express antipathogen or behavioral effectors – has been advanced most in vector control contexts (tsetse, triatomine, mosquitoes) and is conceptually transferable to pest control.^82^, ^83^ Engineered symbionts could be designed to: (i) express repellents or pheromone antagonists in the gut or cuticle; (ii) knockdown genes required for pheromone biosynthesis via double‐stranded RNA delivery; or (iii) express toxins/anti‐reproductive factors. Paratransgenic approaches have demonstrated proof‐of‐concept for pathogen interference and are a promising route to altering behavior.^83^, ^84^ At present, deliberate environmental release of genetically modified microbes for plant protection should be regarded as a highly regulated research‐stage approach rather than a routine operational tool, and any such deployment will require jurisdiction‐specific approval, robust containment, and case‐by‐case ecological risk assessment.

Technical prerequisites and caveats include the following. (i) Stable colonization and vertical/horizontal transmission – for population‐level effects, engineered symbionts should persist in hosts and ideally transmit across generations; otherwise repeated augmentation is required.^82^, ^85^ (ii) Genetic tools and safety mechanisms – CRISPR editing, kill switches and containment strategies must be embedded to limit environmental spread.^59^ antiSMASH and MIBiG enable reverse engineering of BGCs to move biosynthetic pathways into safe chassis, but ecological risk assessments remain essential.^58^, ^59^ (iii) Regulatory and public acceptance – paratransgenic releases face regulatory hurdles and public perception issues; early community engagement and environmental fate studies are imperative.^83^, ^86^

### 4.3 Disrupting symbiotic pathways to break pest communication

Targeted suppression of symbionts or their biosynthetic genes offers a ‘disconnect’ strategy: if a pest requires microbes for pheromone production, eliminating the microbe or blocking its pathway can collapse aggregation or mating signals. Laboratory examples show antibiotic treatment abolishes pheromone emission in some beetles and weevils.^6^ Approaches to disrupt symbiotic pathways include: bacteriophage therapy or specific antimicrobials delivered in bait stations, RNA interference (RNAi)‐based silencing targeted to microbial genes (paratransgenic RNAi), and competitive displacement using benign microbes that outcompete pheromone‐producing taxa.

The advantages and constraints of this method are:

1. Specificity – targeted phage or narrow‐spectrum antimicrobials can minimize non‐target effects, but microbial community redundancy may buffer interventions.
2. Resistance and ecological knock‐ons – selective pressures could select for resistant microbial strains or shifts in microbiomes that restore pheromone production; monitoring and contingency plans are necessary.
3. Ethical and legal considerations – intentional environmental manipulation of microbial communities must meet biosafety standards and community consent.

**Figure 3:**
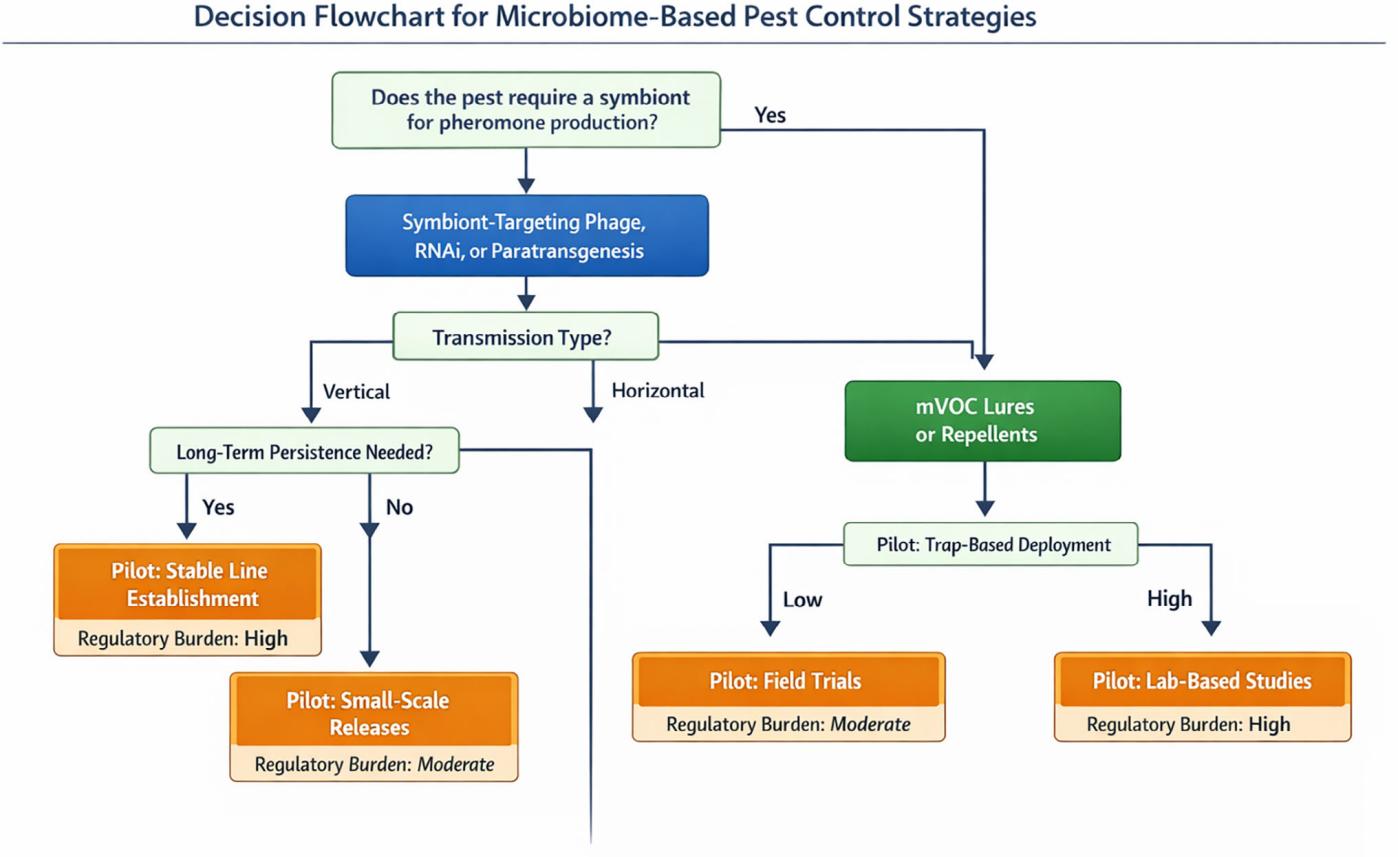
Decision flowchart for selecting microbiome‐based control strategies. The framework compares alternative intervention pathways, including microbial volatile organic compound (mVOC) deployment, engineered bait microbes, paratransgenesis, and symbiont disruption. The flowchart begins with the question: Is there evidence that a symbiont contributes to pheromone production, release, or related signaling in the target pest? If yes, symbiont‐targeting approaches such as phage therapy, RNA interference, or paratransgenesis may be considered; if no, mVOC‐based attractants or repellents are prioritized. Subsequent decision nodes evaluate transmission mode (vertical or horizontal), regulatory complexity, ecological persistence, and operational feasibility. The figure concludes with recommended pilot‐scale actions for each strategy and a qualitative regulatory burden gradient, increasing from contained laboratory validation, through semi‐field testing, to open‐field deployment of engineered microbes or symbiont‐based interventions.

To guide selection among these strategies, Fig. 3 provides a decision flowchart that incorporates key biological, technical, and regulatory considerations.

### 4.4 Integration with IPM and deployment frameworks

Microbiome‐based semiochemical tactics should be integrated, not substituted, into IPM. Practical deployment pathways include:

1. Monitoring enhancement – replace or augment pheromone traps with microbial baits to improve sensitivity (fruit flies, *Drosophila* spp.).^11^
2. Push–pull systems – combine crop‐applied microbial repellents (push) with attractant‐baited trap crops or stations (pull), thereby reducing pest pressure while centralizing mortality.^87^
3. The sterile insect technique (SIT) and autocidal synergies – improve sterile/incompatible insect releases by microbiome supplementation to restore mating competitiveness and pheromone expression in mass‐reared insects (*Wolbachia* programs and SIT interface).^84^, ^88^
4. Sanitation and cultural practices – reduce unwanted microbial fermentations that attract pests by postharvest sanitation and microbiome management.^22^
5. Economic viability and adoption barriers – The practical deployment of microbiome‐based semiochemical tools must confront economic, logistical, regulatory, and social constraints in real‐world IPM systems.

Fermentation baits, despite their high attractiveness, typically require weekly renewal due to depletion of microbial substrates and decline in volatile emission profiles.^11^ This recurring requirement increases material and labor costs, limiting scalability in resource‐constrained agricultural systems. For engineered microbes and paratransgenic symbionts, barriers are substantially more complex. Regulatory approval requires multi‐year environmental risk assessments, demonstration of biosafety, and long‐term field validation, resulting in extended development timelines and high upfront costs that can exceed those of conventional chemical control strategies.^83^ More broadly, regulatory heterogeneity across regions further slows commercialization and increases compliance burden for biocontrol products. Beyond regulatory constraints, farmer and community acceptance remains a decisive determinant of adoption. Empirical studies of symbiont‐based control programs indicate that trust in institutions, perceived risk, and prior familiarity with the technology strongly influence willingness to adopt.^86^ Similar patterns are observed across IPM innovations, where knowledge intensity, uncertainty in field performance, and weak extension support reduce adoption rates despite demonstrated ecological benefits.^87^, ^88^

Together, these constraints indicate that the success of microbiome‐based pest management will depend not only on technical efficacy, but also on early‐stage integration of economic feasibility analyses, regulatory planning, and participatory stakeholder engagement within IPM development pipelines.

### 4.5 Regulatory, biosafety and operational considerations

Any field application of engineered microbes, paratransgenic symbionts, or novel mVOCs requires rigorous biosafety assessment: environmental fate, non‐target toxicity (pollinators, natural enemies), gene transfer risk, and socio‐economic impact must be evaluated. Regulatory frameworks vary by jurisdiction; engagement with regulators early in proof‐of‐concept stages is critical.^83^, ^89^ For chemical mVOCs, toxicity and residue evaluation follow standard pesticide/biopesticide pathways; for live microbes or engineered symbionts, environmental release protocols and post‐release monitoring are mandatory. The design and regulation of engineered bacteria for environmental release involves detailed consideration of performance control, persistence, and biocontainment strategies, as well as adaptation to existing regulatory structures to maximize benefits while mitigating risks.^90^

#### 4.5.1 Practical recommendations

Prioritize field‐validated mVOCs with robust GC–MS/EAG/behavioral pipelines.^11^, ^79^ Use BGC mining (antiSMASH/MIBiG) to identify candidate biosynthetic pathways and transfer to safe chassis for regulated production.^58^, ^59^ For paratransgenesis, demonstrate stable colonization, containment (kill switches), and multi‐year persistence studies before large‐scale releases.^83^, ^91^ Integrate microbial semiochemical tactics with existing IPM tools and monitor non‐target effects continuously.^87^

## 5 CASE STUDIES

Below are rigorous, evidence‐graded case studies across three applied domains – agricultural pests, disease vectors, and stored‐product pests – emphasizing mechanistic evidence, translational status, and development readiness. Each case summarizes the microbe–compound–behavior chain, classifies the evidence strength (correlative /manipulative/genetic/field‐validated), and evaluates pest management readiness.

### 5.1 Agricultural pests: fruit flies and *Drosophila suzukii*

Fruit‐infesting tephritids and drosophilids exploit volatile bouquets produced by yeasts and bacteria on ripening and decaying fruit, and these mVOCs – chiefly ethanol, acetic acid, fusel alcohols and esters – are potent, dose‐ and fermentation stage‐dependent attractants that underlie both long‐standing monitoring tools and recent attract‐and‐kill developments; mechanistic studies show that *Saccharomyces* and non‐*Saccharomyces* yeasts together with Enterobacteriaceae ferment sugars into characteristic volatile blends that are detected by fly olfactory receptors tuned to fermentation cues, and laboratory, engineered‐microbe and field trap studies demonstrate strong, manipulable attraction.^10^, ^11^, ^81^, ^92^ Given the combination of laboratory manipulative experiments and replicated field validation, evidence strength is best described as manipulative + field‐validated: live fermentation baits and single‐strain baiting reliably increase trap captures, metabolic modification of acetic acid bacteria can enhance attractiveness in controlled tests, and mixed‐lure field devices capture more *D. suzukii* than vinegar alone. Translation is therefore high – fermentation‐based lures are already commercial and being optimized – and immediate research and development priorities are identification of minimal, stable synthetic blends mimicking live fermentations, engineering safe production strains or probiotic yeasts for regulated release, and integrating fermentative attractants into attract‐and‐kill or push–pull strategies while addressing non‐target catches and regulatory constraints for live microbe releases.^11^, ^81^, ^92^, ^93^

### 5.2 Vectors: mosquitoes, skin microbiota, and microbial deterrents

Human skin microbiota markedly shape host odor profiles and thereby mosquito host‐seeking: skin bacteria (e.g. *Staphylococcus*, *Corynebacterium* spp.) convert sweat components into short‐chain carboxylic acids, aldehydes and other volatiles that strongly influence *Anopheles*/*Aedes* attraction, and complementary work shows entomopathogenic bacteria (*Xenorhabdus* spp.) produce small‐molecule fractions (putative fabclavines) that act as potent feeding‐deterrents in laboratory assays.^17^, ^65^, ^66^ Evidence ranges from correlative → manipulative (skin microbiome transfer and odor profiling change attractiveness) to laboratory‐validated bioactive compounds, but field translation for microbial repellents or topical applications is still nascent because of safety, approval, and persistence challenges; correspondingly, translational prospects are moderate with two complementary applied routes: (i) baits/traps leveraging microbially derived attractants (e.g. yeast volatiles in sugar baits); and (ii) microbial secondary metabolites as repellents/deterrents – and a longer‐term avenue is paratransgenic/symbiont‐based vector modification (e.g. engineered *Asaia*/*Serratia*), which shows promise but requires robust containment, ecological risk assessment and regulatory pathways.^17^, ^66^, ^83^

### 5.3 Stored‐product pests: grain insects and mVOCs

Postharvest grain environments frequently develop fungal and bacterial growth that emits mVOCs (aldehydes, alcohols, ketones, acids) signaling moisture and substrate suitability; headspace profiling and behavioral assays demonstrate that some primary stored‐product pests (e.g. *Rhyzopertha dominica*, *Sitophilus* spp.) orient to these microbially modified volatiles while many secondary pests show weaker or inconsistent responses, so evidence falls in the correlative → manipulative range depending on species and assay.^22^ Translationally this is moderate: mVOCs can improve early detection and monitoring (lure‐tuned traps) and motivate sanitation strategies that reduce microbe‐driven kairomones, but deployment faces practical constraints (complex volatile backgrounds in storage, dispenser robustness, species‐specificity and possible attraction of non‐target beneficial arthropods), so further work should prioritize identification of the specific behaviorally active mVOCs, gas chromatography‐electroantennographic detection (GC‐EAD) validation, and field formulation/dispenser testing.^22^

### 5.4 Wood‐borers and bark beetles: microbial transformation of host cues

Bark beetles and related xylophagous insects exploit microbial biotransformation of host terpenoids and phenolics: fungal and bacterial symbionts in galleries and beetle microbiomes convert host monoterpenes into oxygenated derivatives (e.g. verbenone, verbenol and other oxygenated monoterpenoids and methylated phenolics) that act as aggregation or anti‐aggregation signals and thereby modulate mass‐attack and host selection behaviors; the biochemical tracing, volatile identification and field syntheses of microbe‐derived pheromone components are well documented, giving mixed but strong mechanistic evidence.^6^, ^67^, ^94^ Where pheromone components are known and synthesizable, translational readiness ranges from moderate to high (monitoring, trap‐tree and semiochemical disruption strategies are already feasible); however, molecular genetic manipulations that conclusively assign biosynthetic steps to specific microbial genes (gene knockouts in symbionts) remain limited, and conceptual strategies such as microbial‐pathway inhibitors or targeted manipulation of symbionts deserve further experimental and regulatory attention.^6^, ^67^

## 6 GAPS, METHODOLOGICAL CHALLENGES, AND FUTURE RESEARCH DIRECTIONS

This section synthesizes the cross‐cutting gaps identified in the evidence above and offers an experimental and translational roadmap to elevate microbiome–semiochemical research to mechanistic and applied maturity.

### 6.1 Principal knowledge gaps

Despite rapid growth in descriptive studies, a rigorous causal chain tying a specific microbial taxon to a defined volatile, and thence to a host behavioral response, remains the exception rather than the rule: many cases report co‐occurrence or correlation between microbes and mVOCs but do not satisfy the gold standard sequence of loss–gain–rescue (axenic/antibiotic removal → loss of compound → loss of behavior → rescue by single‐strain recolonization or heterologous expression) that provides mechanistic proof.^13^, ^14^ Closely allied is the limited genetic validation of BGCs: modern genome and metagenome mining (antiSMASH, MIBiG and related tools) can nominate candidate BGCs, but functional confirmation by gene deletion, CRISPR perturbation or heterologous expression that links BGC → product → host behavior remains rare, hampering targeted engineering or disruption approaches.^58^, ^59^, ^95^ Third, context‐dependency is profound: diet/substrate, developmental stage, host physiology and environmental microbiomes strongly modulate microbial composition and volatile output, and short‐term laboratory assays frequently fail to predict field outcomes – longitudinal, multi‐site field validation is comparatively scarce. Fourth, safety, non‐target impacts and regulatory pathways for the release of live or engineered symbionts (paratransgenesis) are underdeveloped: environmental fate, horizontal gene transfer potential and effects on beneficial arthropods require explicit, early risk assessment and community engagement prior to releases.^83^, ^96^ Together, these gaps limit reliable translation from discovery to operational pest management tools and argue for experimental workflows that move beyond correlative descriptions to reproducible mechanistic demonstrations.

### 6.2 Methodological recommendations (practical, stepwise)

**Figure 4:**
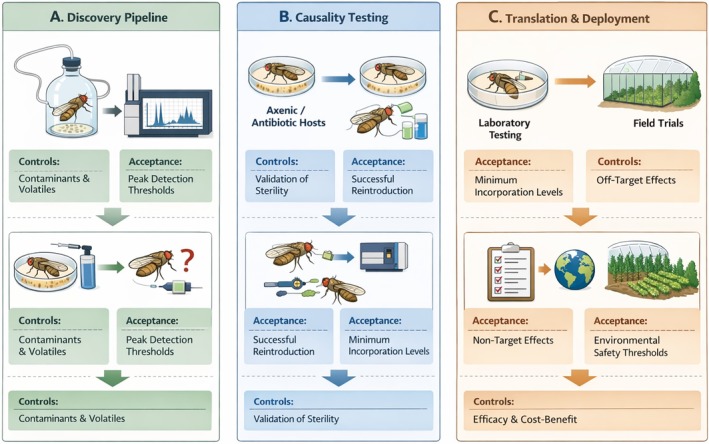
Methodological pipeline and recommended assays. (A) Laboratory pipeline for discovery [headspace sampling → two‐dimensional gas chromatography (GC × GC) → candidate compound identification (ID) → electroantennography/single‐sensillum recording (EAG/SSR) screening]. (B) Causality testing (axenic/antibiotic hosts → recolonization → isotope tracing → microbial gene knockout/heterologous expression). (C) Translation and deployment (laboratory → small‐scale field trials → regulatory environmental risk assessment → scale‐up and IPM integration). Each panel lists critical controls and acceptance criteria (e.g. minimum effect sizes, non‐target thresholds).

To close the gaps and generate translationally useful knowledge, we recommend a staged, evidence‐driven pipeline that couples high‐resolution chemistry, causality testing, genetic validation, field relevance and regulatory foresight. (i) Discovery and chemical validation – pair culture‐dependent headspace sampling with culture‐independent VOC‐guided metagenomics/metatranscriptomics and use HS‐SPME coupled to GC–MS and GC × GC‐quadrupole Mass Spectrometer (qMS) (or comparable high‐resolution platforms) for sensitive fingerprinting and quantification of candidate mVOCs^97^. (ii) Causality testing – implement axenic/antibiotic manipulations, single‐strain recolonization or defined consortia, and stable isotope (e.g.^13^C) precursor tracing to demonstrate microbe → metabolite flux and show concordant changes in electrophysiology (EAG/SSR) and choice assays.^14^, ^98^ (iii) Genetic validation – mine genomes/metagenomes for candidate BGCs using antiSMASH/MIBiG, confirm expression with metatranscriptomics/proteomics, and perform targeted gene deletions or heterologous expression (in safe production chassis) to demonstrate loss/gain of volatile production and behavioral effect.^58^, ^59^, ^95^ (iv) Field relevance and delivery – progress promising blends or live baits into replicated multi‐site, seasonal field trials that quantify efficacy, persistence and non‐target catches and compare live fermentation baits *versus* defined synthetic blends and protected dispensers. (v) Regulatory and biosafety pipeline – integrate environmental risk assessments (non‐target toxicity, gene‐flow modeling), design biological containment (auxotrophies, kill switches), and execute stakeholder and regulatory engagement early and iteratively.^83^, ^96^ Following this pipeline will prioritize mechanistic certainty while accelerating safe, reproducible translation. Figure 4 summarizes this recommended multi‐layered pipeline in three panels, highlighting key assays, controls, and acceptance criteria at each stage to facilitate rigorous progression from discovery to deployment.

### 6.3 Emerging technological priorities

A pragmatic roadmap for accelerating mechanistic and applied maturity should prioritize: (i) single‐cell and long‐read metagenomics to recover contiguous genomes and full BGCs from unculturable symbionts and enable accurate binning and functional annotation, thereby improving candidate selection^99^, ^100^; (ii) advanced metabolomics integrated with stable isotope resolved metabolomics to trace precursor flow from microbe to volatile and host tissues and to quantify production dynamics *in situ*; (iii) synthetic biology and heterologous BGC reconstruction in well‐characterized, safe production strains to enable predictable,^101^ scalable mVOC production for both field dispensers and biochemical validation; (iv) electrophysiology and odorant receptor deorphanization (heterologous odorant receptor expression, SSR/EAG) to map receptor–ligand pairs for mVOCs, enabling reverse chemical ecology optimization and tailored receptor‐based biosensors; and (v) socio‐ecological and stakeholder research (structured engagement frameworks, acceptability studies, transparent risk communication) to address public and regulatory concerns about live microbe releases and engineered symbionts – all of which together form the technical and social scaffolding needed for safe, effective translation.^95^, ^96^, ^98^, ^102^, ^103^

### 6.4 Representative examples and evidence priorities

To summarize the current state of the field and to inform future research directions, Table 3 presents a set of representative, well‐documented examples of microbial contributions to insect semiochemicals. Cases are grouped by semiochemical class and, for each entry, detail the microbial source, host insect, associated behavioral or ecological function, proposed mechanism of action, and the primary supporting reference. Together, these examples capture the breadth of evidence currently available.

**Table 3:** Microbial contributions to insect semiochemicals

Semiochemical (Class)	Microbial source	Insect host	Behavioral/ecological role	Mechanism	References
Sex pheromones					
Pyrazines: 2,3,5‐trimethylpyrazine (TMP) and 2,3,5,6‐tetramethylpyrazine (TTMP)	*Bacillus* spp. (gut/rectal)	*Bactrocera dorsalis*	Female attraction to males	Produced *de novo* from glucose/threonine; antibiotic depletion reduces pheromone; evening emission peak	35
4,8‐Dimethyldecanal (4,8‐DMD)	*Escherichia coli* (gut bacterium)	*Tribolium castaneum* (red flour beetle)	Male attractiveness to females; mating communication	Gut bacteria regulate pheromone synthesis indirectly by upregulating TcFAS1; RNAi knockdown reduces 4,8‐DMD	33
Aggregation pheromones/cues					
Guaiacol	*Pantoea agglomerans*, Enterobacteriaceae	*Schistocerca gregaria*	Swarming, cohesion	Gut bacteria metabolize plant‐derived precursors in feces	104
Volatile carboxylic acids	Gut bacteria (aerobes)	*Blattella germanica*	Aggregation	Fecal bacterial VOCs attract conspecifics; microbiota essential	14
2,6,10,14‐Tetramethylheptadecane and heptacosane	*Acinetobacter guillouiae* and other gut Proteobacteria	*Trigonorhinus* sp.	Aggregation, mate attraction	Gut bacteria essential for long‐chain alkane biosynthesis; recolonization restores signal	36
Pheromone/CHC mix (ML, MM, MP, cVA, CHCs)	Frass chemical signature reflecting gut metabolism (potential microbiome contribution)	*Drosophila melanogaster*	Feeding stimulation and aggregation at food sources	Frass contains pheromone compounds mirroring adult profiles, acting as olfactory cues; detection via ORs and possibly taste receptors	105
Verbenone	Gut bacteria (e.g. *Serratia*, terpene‐metabolizers)	*Dendroctonus valens*	Aggregation regulation, host selection (dose‐dependent)	Bacterial biotransformation of host monoterpenes (e.g. *α*‐pinene) into verbenone	64
Verbenone modulation (microbiome‐dependent)	Gut microbiota community (pH‐sensitive)	*Dendroctonus valens*	Aggregation and host colonization	Gut pH alters microbial composition, reducing verbenone‐producing bacteria and pheromone output	106
Kairomones/Attractants					
Honeydew VOCs	*Staphylococcus*, *Acinetobacter*, *Pseudomonas* spp.	Aphids and predators (ladybirds, syrphids)	Predator attraction, oviposition site selection	Bacterial metabolism of honeydew sugars produces attractive VOCs	107
Honeydew volatiles	Five cultivable bacteria from *Aphis gossypii* honeydew	*Hippodamia variegata*	Predator attraction, prey location	Bacteria produce 1‐heptanol, 2‐ethyl‐1‐hexanol, 2‐phenylethanol, and 2‐methyl‐1‐propanol	55
3‐Hexenyl acetate	*Citrobacter* sp. (*CF*‐BD strain)	*Bactrocera dorsalis*	Attraction, oviposition	Produced in bacterially colonized fruits; detected via ovipositor sensilla	108
Acetic acid, acetoin, 2,3‐butanediol	Yeasts (*Hanseniaspora*, *Saccharomyces*)	*Drosophila suzukii* and *D. melanogaster*	Long‐range attraction, foraging, oviposition	Yeasts ferment substrates *de novo*, producing guiding volatile blends	109
Floral volatiles (terpenoids, esters)	Floral microbiota (yeasts and bacteria)	Pollinators (bees, parasitoids)	Modulation of visitation and foraging	Microbes add to or modify floral VOC blends	110
Nectar VOCs (alcohols, esters)	Nectar yeasts (*Metschnikowia* spp.)	*Culex pipiens*	Nectar foraging, pollination	Yeast‐produced volatiles attract mosquito visitors	111
Nectar VOC modulation	Nectar yeasts (*M*. *gruessii*, *M*. *reukaufii*)	*Aphidius ervi*	Foraging preference	Nectar composition and yeast fermentation alter VOC profiles, affecting parasitoid attraction	12
Skin VOCs (Lactic Acid, Butyric Acid)	Human skin microbiota (*Staphylococcus*, *Corynebacterium*)	Mosquitoes (*Aedes aegypti*)	Host‐seeking, attraction	Bacterial metabolism of skin compounds produces attractive VOC blends	75
Plant VOCs induced by insect‐associated bacteria (Indirect cue)	Insect‐associated bacteria of *Oulema melanopus*	*Oulema melanopus* and host wheat	Modulation of plant VOC emissions; potential attraction of natural enemies	Insect‐associated bacteria enhance diversity and abundance of herbivore‐induced plant VOCs	31
Contact pheromones					
Cuticular hydrocarbons profile modulation [Contact/sex pheromone: cuticular hydrocarbons (CHCs)]	Gut microbiota (commensal bacteria, e.g. *Lactobacillus* spp.)	*Drosophila melanogaster*	Mate choice and assortative mating – flies preferentially mate with others whose microbiota shapes CHC profile similarity	Gut microbiota manipulation (diet/antibiotics) alters CHC pheromone profiles; loss of microbes abolishes mating preference and bacterial reintroduction restores it	13
CHCs profile modulation	Endosymbiotic bacterial community (e.g. *Wigglesworthia*, *Sodalis*, *Wolbachia* influenced)	*Glossina morsitans morsitans* (Tsetse fly)	Mate choice and courtship success – flies with intact microbiota were preferred over symbiont‐depleted individuals	Antibiotic disruption of symbiotic bacteria altered CHC profiles (↓15,19,23‐trimethyl‐heptatriacontane), reducing mate preference for microbiota‐depleted flies	112
Antifeedants/deterrents (microbial VOCs)					
Antifeedant VOCs	Gut bacterial isolates (*Rahnella aquatilis*, *Brevundimonas nasdae*)	*Hylobius abietis* (pine weevil)	Feeding deterrence on host seedlings	Gut bacteria produce VOCs (e.g. 2‐phenylethanol, 2‐methoxyphenol, 3‐methyl‐1‐butanol, 1‐octen‐3‐ol, 3‐octanone, dimethyl disulfide) that strongly deter feeding; 2‐phenylethanol shows dose‐dependent antifeedant effect in bioassays	113

Abbreviations: CHC, cuticular hydrocarbon; OR, odorant receptor; RNAi, RNA interference; VOC, volatile organic compound; cVA, 11‐cis‐ vaccenyl acetate; ML, Methyl Laurate; MM, Methyl Myristate; MP, Methyl Palmitate.

The entries span a continuum of evidentiary strength, ranging from high‐confidence systems supported by manipulative loss–gain–rescue experiments, coupled chemical and behavioral validation, and, in some instances, genetic confirmation, to more preliminary or correlative cases. Recent *Drosophila* work further extends this spectrum to metabolite‐driven microbiome–host signaling.^114^ The former represent systems that are mature enough to support lure or repellent optimization and, in some cases, progression to field‐based evaluation. By contrast, emerging systems will require progression through the staged framework outlined above – discovery, causal validation, genetic confirmation, and multi‐site field testing – before they can be reliably translated into applied or predictive contexts.

## 7 CONCLUSIONS

Microbiome–semiochemical interactions represent a frontier with both fundamental and applied promise. A growing number of studies show that microbes – in the gut, on the cuticle, in frass, and in the environment – can produce or modulate semiochemicals that drive aggregation, mating, host selection and other behaviors of pest insects. Several translational pathways are already apparent: fermentation lures for fruit flies, microbial metabolites as novel repellents for vectors, and the conceptual application of microbiome engineering or symbiont disruption for behavior modification.

However, the field still lacks the systematic mechanistic evidence required to scale many concepts to reliable, regulatory‐compliant pest control products. To progress, researchers must adopt multi‐omics, functional genetics, isotope tracing, electrophysiology and rigorous field validation as a standard pipeline. Gene‐to‐molecule chains (BGC → VOC → receptor → behavior) are the gold standard that will permit targeted engineering, safe deployment, and integration into IPM. When combined with careful biosafety assessment and stakeholder engagement, microbiome‐based semiochemical innovations could provide powerful, sustainable tools for pest management.

## FUNDING INFORMATION

This research was supported by the National Natural Science Foundation of China (32472644) and the Guizhou Provincial S&T Innovation Platform Research Project (Qiankehe Pingtai CXPTXM[2025]‐026).

## CONFLICT OF INTEREST

The authors have no conflict of interest to declare.

## Data Availability

No data were used or generated during the course of this study.
